# AQbD Approach Applied to NIR in a Complex Topical Formulation: Bifonazole as Case Study

**DOI:** 10.3390/pharmaceutics17070835

**Published:** 2025-06-26

**Authors:** Lucas Chiarentin, Vera Moura, Alberto A. C. C. Pais, Carla Vitorino

**Affiliations:** 1 Faculty of Pharmacy, University of Coimbra, Pólo das Ciências da Saúde, Azinhaga de Santa Comba, 3000-548 Coimbra, Portugal; lczenith@gmail.com; 2 Laboratórios Basi Indústria Farmacêutica S.A., Parque Industrial Manuel Lourenço Ferreira, Lote 15, 3450-232 Mortágua, Portugal; 3 Basinnov Life Sciences, Unipessoal LDA, Avenida José Malhoa, Ed. Malhoa Plaza, n° 2, 3° piso, Escritório 3.7, 1070-325 Lisboa, Portugal; vera.moura@basinnov.pt; 4 Coimbra Chemistry Centre, Institute of Molecular Sciences—IMS, Chemistry Department, University of Coimbra, 3000-535 Coimbra, Portugal; pais@qui.uc.pt

**Keywords:** FT-NIR, RP-HPLC, complex formulation, analytical quality by design (AQbD)

## Abstract

**Background:** A key challenge in modern pharmaceutical research is developing predictive models for drug formulation behavior. Since permeability is closely linked to molecular properties, considering a broad of characteristics is essential for building reliable predictive tools. Near-infrared spectroscopy (NIR), a non-destructive, non-invasive, and chemically specific method, offers a powerful alternative to current gold-standard methods approved by regulatory agencies. **Objectives:** This study aims to apply a partial analytical quality by design (AQbD) approach to enhance the understanding and development of NIR and RP-HPLC methodologies. **Methods:** The employment of NIR with multivariate data analysis enabled the establishment of chemometric models for the classification and quantification of bifonazole (BFZ) in cream formulations. **Results:** An analytical target profile (ATP) was defined to guide the selection of critical method variables and support method design and development activities. Risk assessment was carried out using an Ishikawa diagram. For the RP-HPLC method, key performance parameters such as peak area, theoretical plates, tailing factor, and assay were evaluated, while NIR spectra and BFZ concentration were considered for method performance. The quantification models enabled the accurate determination of BFZ content, yielding results of 8.48 mg via NIR and 8.34 mg via RP-HPLC, with an RSD of 1.25%. **Conclusions**: These findings demonstrate the robustness and reliability of the models, making them suitable for routine quality control of BFZ formulations. Future research should aim to explore its use for monitoring permeation dynamics in real time and integrating it into regulatory frameworks to standardize its application in pharmaceutical quality control and formulation development.

## 1. Introduction

Bifonazole [1-[[1,1′-biphenyl)-4-phenylmethyl]-1*H*-imidazole) (BFZ) is a broad-spectrum antifungal agent belonging to the class of imidazole derivatives. BFZ is utilized for the treatment of skin or mucosal mycoses. Its topical administration enables an intensive drug retention in the epidermis, thus treating infections caused by dermatophytes, yeasts, molds, and fungi [1,2,3,4]. The available formulations of BFZ in the market include dusting powders and semisolids such as creams and lotions [5,6,7,8]. The quality control of BFZ formulations, which is essential for effective treatment, is typically performed using high-performance liquid chromatography (HPLC) [9,10], as described in the European Pharmacopeia (EP) [11], the United States Pharmacopeia (USP), and the British Pharmacopoeia (BP) [12]. Regulatory authorities, such as the European Medicines Agency (EMA), have also encouraged alternative analytical techniques, including near-infrared spectroscopy (NIR), to complement traditional methods [13].

NIR is an analytical procedure, often requiring chemometric statistical analysis, with broad and versatile applications in pharmaceutical analysis. These applications include the identification, qualification, and assay of starting materials, intermediates, and finished products, as well as the verification of physicochemical properties. The effective use of NIR relies on a thorough understanding of the product. The application of analytical quality by design (AQbD) (as outlined in ICH Q14) is considered appropriate, with the extent of method development and/or validation tailored to the control strategy [13,14]. Additionally, the processes can be modeled mathematically, with diffusion models of varying complexity providing valuable insights [15].

Chemometrics methods are essential for extracting meaningful and useful information from large datasets. To achieve reliable and accurate calibration models, data must often be pre-processed if necessary [16]. A variety of chemometrics techniques, encompassing mathematical and statistical methods, have been developed to handle data effectively at different stages. Chemometrics is primarily applied in four steps: data acquisition, data pre-processing, classification or regression model building, and model validation [17,18,19].

To improve spectral resolution and enhance the signal-to-noise ratio, several spectral derivation and smoothing techniques have been proposed, including Savitzky–Golay (SG) polynomial derivative filters and the Norris–Williams algorithm, which help eliminate baseline variations and resolve overlapping peaks. Additionally, scatter-correction pre-processing methods such as standard normal variation (SNV), detrend (DT), and multiplicative scatter correction (MSC) are widely used to minimize additive effects in signal intensity [20].

This approach offers an analysis of the current state of the art in monitoring drug products and dermal penetration of complex topical formulations in vivo, as well as the regulatory requirements of international guidelines.

With the advancement of cheminformatics, numerous quantitative structure–activity relationships (QSAR) modeling techniques have been developed, including support vector machine (SVM), artificial neural networks (ANNs), multiple linear regression (MLR), principal component regression (PCR), and partial least squares (PLS) regression [14]. Among these, the PLS method enables the linear correlation of multiple observations and *X* variables with one or more *Y* variables, making it a powerful tool for complex datasets [21,22].

The development and implementation of an NIR procedure is an iterative, ongoing process that aligns well with the application of lifecycle concepts. Standardization protocols for evaluating drug measurements in pharmaceutical products are essential to ensure the consistency, reliability, and comparability of results across different formulations and experimental conditions.

**Figure 1:**
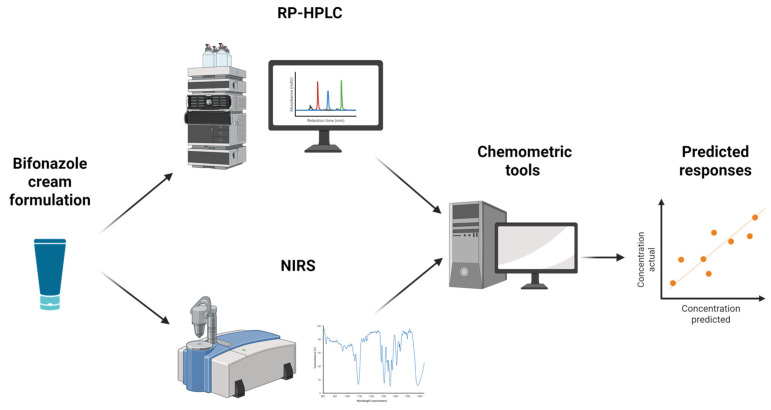
Workflow for determining bifonazole in cream formulation through NIR. Created in https://BioRender.com.

The main scope of this research was to develop RP-HPLC and NIR methodologies combined with multivariate analysis for the quantification of BFZ in pharmaceutical formulations, under the umbrella of AQbD (Figure 1). AQbD aims to optimize partial or all stages of the analytical procedure life cycle by identifying and controlling critical method variables (CMVs) and risk factors throughout the entire analytical protocol.

## 2. Materials and Methods

### 2.1. Materials

Bifonazole, sorbitan stearate, polysorbate 60, cetyl palmitate, cetostearyl alcohol, 2-octyldodecanol, and benzyl alcohol were supplied by Laboratórios Basi Indústria Farmacêutica S.A. (Mortágua, Portugal). The hydrophobic PTFE filter was obtained from Filter Lab, Barcelona, Spain. All other reagents and solvents were of analytical or HPLC grade.

### 2.2. Preparation of Bifonazole Cream Formulation

Bifonazole cream formulations were prepared using a conventional method with an Ultra-Turrax X 10/25 (Ystral GmbH, Dottingen, Germany) equipment. The continuous and dispersed phases were prepared separately and heated to 68 ± 2 °C, after which the active pharmaceutical ingredient (API) was dissolved in the dispersed phase. The final cream formulations were stored at 20–25 °C, with batches produced at 0.5 kg. These formulations were utilized for method development purposes. Please note that, due to confidentiality, the complete manufacturing process cannot be disclosed.

### 2.3. Analytical Methods Development

AQbD adopts a science-driven and systematic approach to analytical procedure development, encompassing all stages of a method’s lifecycle—from design and validation to transfer and routine use. By closely integrating these stages, AQbD provides a comprehensive framework for mitigating the risk of the method failure and ensuring consistent performance throughout the lifecycle [23,24]. AQbD utilizes risk-based strategies and statistical methodologies to guide the development of analytical procedures. Central to AQbD is knowledge and risk management, enabling the design and understanding of analytical method performance from the earliest stages of development. Effective method design requires a systematic analysis of the factors influencing method quality, which involves a series of sequential and interconnected steps. A partial AQbD approach is described in detail in the following sections for the development of RP-HPLC and NIR methods [25,26].

#### 2.3.1. Risk Assessment

The AQbD workflow begins by defining the target measurement and establishing analytical target profile (ATP), which outlines key method quality features that should ideally be met to ensure both efficiency and reliability. Within the ATP, potential critical analytical attributes (CAAs) were identified as the essential parameters that must remain within specified limits to guarantee the desired method quality.

Prior to optimization of chromatographic and NIR analysis conditions, the most crucial step is risk assessment. It helps in computing the probability of the risk and failure of the parameters that have major effect on the selected CAAs which are known as CMVs. The CMVs were achieved by outlining their relationship with CAAs and conceptualizing the Ishikawa diagram. An Ishikawa diagram was constructed based on prior knowledge and literature, considering the following categories: equipment, environment, method, measurement, analyst, material, sample, and data [27].

#### 2.3.2. RP-HPLC

##### Instrumentation and Chromatographic Conditions

The RP-HPLC analysis of BFZ was performed using a Shimadzu LC-10AD system, equipped with a quaternary pump (LC-10AD), an autosampler (SIL-10ADVP), a CTO-10AVP oven, and a CBM-20 A detector. A Merck C18 column (Torrance, CA, USA), with 5 µm particle size, 4.6 µm internal diameter, and 150 nm length, was used, with the column oven maintained at 30 °C. Chromatographic analysis was conducted in isocratic mode and the mobile phase consisted of a mixture of phase A (a buffer solution at pH 3.2 prepared by mixing 2.0 mL of phosphoric acid with 980 mL of ultrapure, adjusted to pH 3.2 ± 0.05 with triethylamine and dilute to 1000 mL with ultrapure) and phase B (acetonitrile) in a 50:50 *v*/*v* ratio. The flow rate was set at 0.8 mL/min, BFZ detection was performed at 210 nm, and 5 µL of the sample was injected.

##### Preparation of Standard Solutions for Drug Formulations

BFZ (10 mg in 10 mL) stock solution was prepared in acetonitrile (ACN) and sonicated to guarantee a complete dissolution. Calibration standards (75, 112.5, 150, 187.5, and 225 µg·mL^−1^) were prepared by sequential dilution of stock solution with ACN and filtered through 0.22 µm PTFE filters prior to injection. The calibration curve was constructed by linear regression of the average peak area against concentration.

##### Preparation of Sample Solutions for Drug Formulation

The BFZ 10 mg/g cream was diluted with a BFZ cream placebo in order to obtain concentrations of 2.5, 3.75, 5.0, 6.25, and 7.5 mg/g. These preparations were analyzed using NIR and RP-HPLC methods. For RP-HPLC analysis, solutions were prepared by weighing an appropriate amount of each concentration and diluting it with ACN to achieve a final concentration of 150 µg·mL^−1^. The mixtures were vortexed for 1 min, sonicated for 10 min, and subsequently filtered through 0.22 µm PTFE filters prior to analysis.

#### 2.3.3. NIR

##### Instrumentation and NIR Conditions

NIR spectra were collected in transflectance mode using a Spectrum 400 FT-IR and FT-NIR spectrometer (PerkinElmer, Rodgau, Germany), fitted with an InGaAs detector and an optic fiber probe with 1 mm path length. The instrument resolution was specified at 2 cm^−1^. Each spectrum was acquired by averaging 32 scans over the wavenumber range 4100–10,000 cm^−1^, and background spectra were obtained in air.

##### Multivariate Data Treatment

The BFZ mass fraction values were modeled from the NIR spectra using partial least-squares (PLS) regression. The optimal number of latent variables was determined through the leave-one-out cross-validation method [28]. PLS, a supervised method, resolves the bilinear matrix (X) into components (latent variables) while emphasizing variance correlated with the response variable (Y). This approach enables the development of predictive models that can replace the slower RP-HPLC method. PLS aims to regress the result towards a given direction (the reference method) [29,30].

The PLS approach is particularly useful when dealing with more explanatory variables than observations or highly correlated variables. The fitting model algorithm employed was nonlinear iterative partial least squares (NIPALS) using fast SVD. The optimal number of factors was determined through van der Voet T^2^ test and cross-validation, with leave-one-out chosen as the cross-validation method [30].

The predicted residual sum of squares (root mean PRESS) for a factor is the square root of the average PRESS values across all responses. The square root of PRESS provides an easily interpretable error measure and generally decreases with additional mode factors [30].

Variable importance in projection (VIP) scores assess the importance of each predictor variable in the model. A VIP score greater than 1 indicates an important variable for predicting the response. Models with more important variables generally exhibit better performance [30].

## 3. Results and Discussion

The discussion of the results is divided into three parts. First, the results regarding the analytical target profile (ATP) and risk assessment for both methodologies such as RP-HPLC and NIR methodologies are addressed. Afterwards, the results concerning drug quantification in drug products are discussed. To conclude, the predicted response for drug product quantification is explained by the multivariate model.

Within the analytical quality by design scope, the definition of ATP were described to obtaining the optimal method performance [25]. Taking into account such considerations and preliminary studies, the ATP was predefined for the RP-HPLC and NIR methods (Table 1).

Table 1 presents the ATP developed for quantifying BFZ in a semisolid cream formulation using the RP-HPLC and NIR methods. The ATP identifies BFZ as the analyte of interest at a concentration of 10 mg/g in a development-stage product. The justifications for choosing both analytical techniques include their suitability for evaluating semisolid formulations and ensuring method performance in early-stage product development. RP-HPLC, a well-established chromatography method, offers high specificity through retention time and peak resolution metrics. In contrast, NIR provides a rapid, non-destructive alternative by leveraging spectral data to predict concentrations. Critical analytical attributes for each method are outlined, emphasizing performance indicators such as plate number and spectral accuracy.

**Figure 2:**
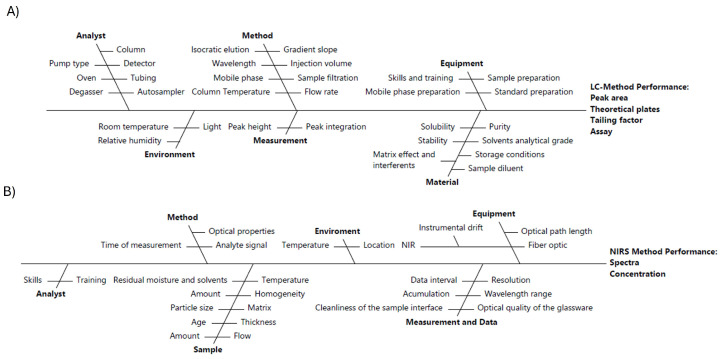
Ishikawa diagram depicting cause-and-effect relationships on the potential CAAs of RP-HPLC (**A**) and NIR (**B**) method.

To further the CMVs, a risk assessment was conducted using an Ishikawa diagram, allowing for the identification and prioritization of parameters that could affect method performance and adherence to the ATP [31]. Figure 2 showcases the Ishikawa diagram proposed by the authors, designed to support analytical target development for both the RP-HPLC and NIR methods.

Given the multitude of factors that can affect chromatographic and spectroscopic method development, narrowing down to the most critical method parameters for experimental evaluation can be challenging. A structured risk assessment approach facilitates not only the identification of CMVs, but also their prioritization based on potential impact. The Ishikawa diagram was constructed using a combination of prior experimental data and theoretical knowledge, in alignment with the science- and risk-based principles outlined in the ICH Q14 guideline. The diagram illustrates potential sources of variability and CAAs associated with RP-HPLC and NIR methods. For RP-HPLC, key performance indicators such as peak area, theoretical plates, and tailing factor are considered, while for NIR, spectral quality and concentration accuracy are emphasized. The diagrams map out cause-and-effect relationships that may influence these attributes, thereby supporting the development of robust analytical procedures. In the case of RP-HPLC, influential factors include equipment components (e.g., column, detector), method parameters (e.g., mobile phase composition, flow rate), and analyst-related variables. For NIR, critical factors encompass sample presentation, environmental conditions, and instrument configuration (e.g., optical path length). By visually organizing these variables, the Ishikawa diagrams aid in defining control strategies and enhancing understanding of method performance variability during development.

### Scrutinizing Drug Product Quantification

**Figure 3:**
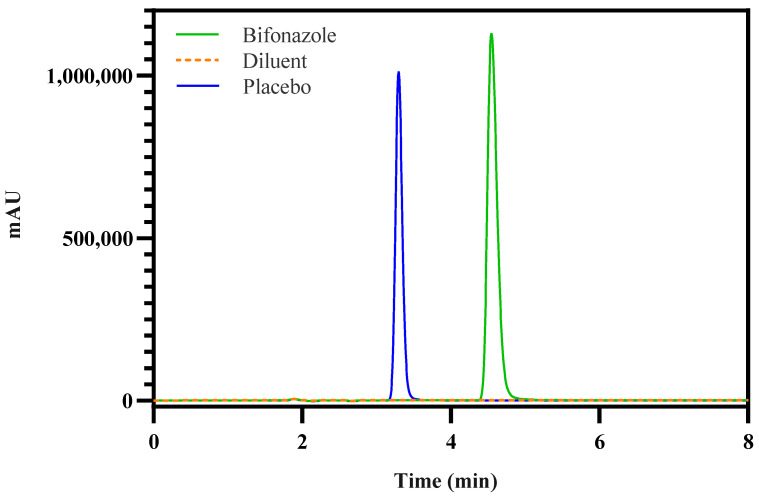
Representative chromatograms of 150 µg·mL^−1^ standard solution of bifonazole (green solid line), diluent (blue solid line), and placebo (orange dashed line).

The RP-HPLC method was developed for bifonazole quantification in drug products, with a focus on CMVs such as flow rate, and mobile phase ratio (in % of ACN), as guided by an Ishikawa diagram. These variables were studied for their impact on CAAs, including peak area, theoretical plates, tailing factor, and assay accuracy. Chromatographic conditions were established following preliminary studies, and the method’s selectivity was confirmed by injecting diluent and placebo solutions, neither of which exhibited a retention time corresponding to bifonazole. Apart from the preservative peak in the placebo solution, no other peaks are presented in the chromatogram, implying that the method is specific to bifonazole (Figure 3). The regression equation for the calibration curve considering five bifonazole standards (75–225 µg·mL^−1^) yielded a regression with a determination coefficient of 0.99, indicating excellent linearity.

The developed method was used to determine the content of BFZ samples described in Section 2.3. and the same samples were analyzed using the developed NIR method. A multivariate calibration approach in chemometrics was applied to establish the relationship between NIR wavelengths and the property of interest, creating a predictive model. This model was subsequently used to predict the same properties from NIR spectra of unknow samples for quantitative analysis [32].

The multivariate approach employed to correlate bifonazole concentration with NIR spectra was partial least squares (PLS), utilizing leave-one-out cross-validation [33]. This method constructs linear models based on factor-based combinations of explanatory variables (*X*s), enhancing predictive accuracy and robustness. These factors are designed to maximize the covariance between the *X*s and the responses (*Y*s), thereby leveraging the correlations between them to uncover underlying latent structures. This approach balances the dual objectives of explaining both response variation and predictor variation. PLS is particularly advantageous when the number of *X* variables exceeds the number of observations or when the *X* variables exhibit high collinearity [30].

The nonlinear iterative partial least squares (NIPALS) algorithm was used for the analysis. NIPALS operates by extracting one factor at a time. Let *X* = *X*_0_ represent the centered and scaled matrix of predictions, and *Y* = *Y*_0_ the centered and scaled matrix of response values. The algorithm begins by defining a linear combination *t* = *X*_0_*w* of the predictors, where *t* is the score vector and *w* is its associated weight vector [30]. The PLS method then predicts both *X*_0_ and *Y*_0_ through regression on *t*, as described in Equations (1) and (2):

(1) X^0=tp′,where p′=t′t−1t′X0

(2) Y^0=tc′,where c′=t′t−1tY0

The vectors *p* and *c* are referred to as *X*- and *Y*-loadings, respectively. The specific linear combination *t* = *X*_0_*w* is chosen to maximize the covariance *t*′*u*, where *u* = *Y*_0_*q* is a linear combination of the responses. Moreover, the *X*- and *Y*-weights, *w* and *q*, are proportional to the first left and right singular vectors of the covariance matrix *X*_0_′*Y*_0_. Alternatively, they correspond to the first eigenvectors of *X*_0_′*Y*_0_*Y*_0_′*X*_0_ and *Y*_0_′*X*_0_*X*_0_′*Y*_0_ respectively [30]. This describes the extraction of the first PLS factor.

The second factor is derived similarly, using *X*- and *Y*-residuals obtained after the first factor is removed, as defined in Equations (3) and (4):

(3) $$X_{1}=X_{0}-{\hat{X}}_{0}$$

(4) $$Y_{1}=Y_{0}-{\hat{Y}}_{0}$$

These residuals, also known as the deflated *X*- and *Y*-blocks, are used to extract subsequent factors. The process of calculating a score vector and deflating the data matrices is repeated for as many factors as required [30].

The model was validated using leave-one-out cross-validation. The cross-validated explained variance ($Q_{y}^{2}$) in *y*, expressed as a percentage, was calculated using Equation (5):

(5) $$Q_{y}^{2}=\left[\frac{{\left(y-ŷ\right)}^{T}\left(y-ŷ\right)}{y^{T}y}\right]\times 100$$

In Equation (5), *y* denotes the vector of mean-centered quality parameter values for each sample, while *ŷ* contains the corresponding mean-centered model predictions. Each element of *ŷ* was derived by projecting the respective sample onto a model calibrated with the remaining samples (leave-one-out cross-validation) [34]. This validation approach ensures model robustness and quantifies its predictive accuracy.

**Figure 4:**
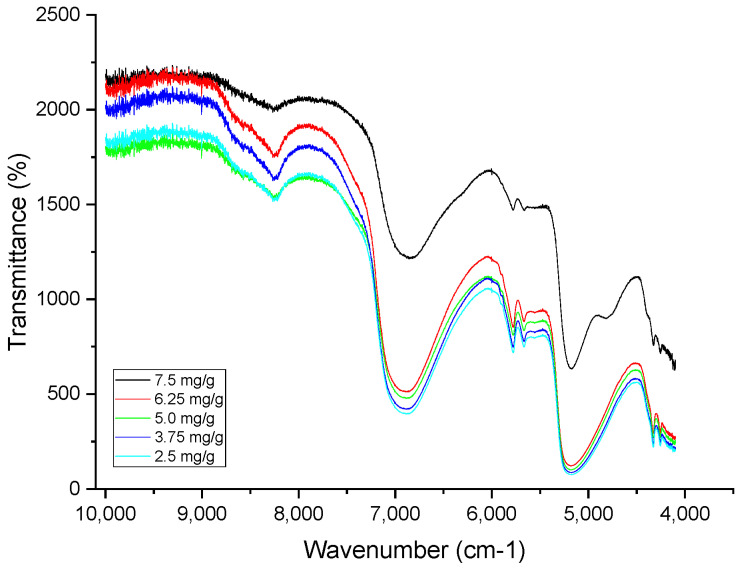
NIR spectra from 10,000 to 4100 cm^−1^ from the BFZ product.

In JMP^®^ (JMP Pro 17), data can be partitioned into sets prior to modeling to minimize the risk of overfitting and to select an optimal predictive model. This approach divides the original dataset into three subsets: training, validation, and test sets. The training set is used to estimate the model parameters. The validation set is employed during the model-fitting process to fine-tune the parameters and select a model with strong capability. Finally, the test set is used to independently assess the performance of the fitted model [35]. Figure 4 represents the main spectra obtained from the NIR.

**Figure 5:**
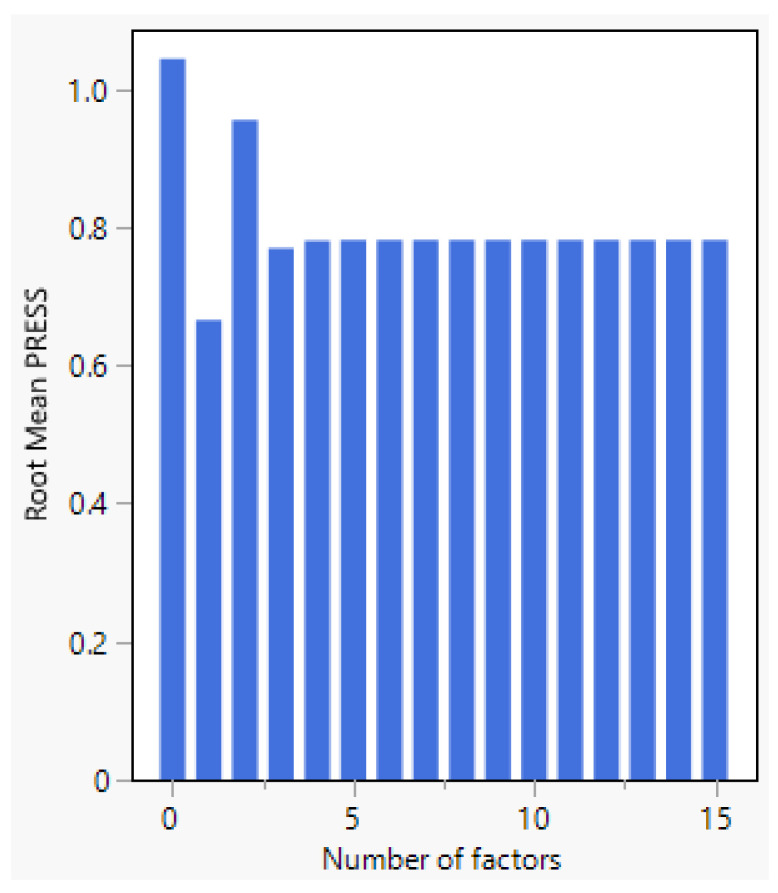
Root mean PRESS for different numbers of factors for drug product quantification.

**Figure 6:**
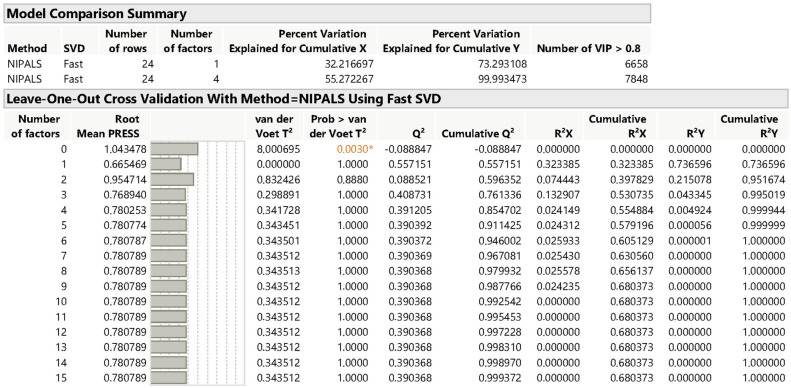
Leave-one-out cross-validation report with method NIPALS using fast SVD for drug product quantification. Key: * *p*-value < 0.05.

The number of factors in a PLS model represents the number of components or latent variables extracted. Figure 5 shows that the root mean PRESS (predicted residual sum of squares) is 0.66547, and with the minimized number of factors being one. The van der Voet T^2^ test (Figure 6) evaluates whether models with different numbers of factors differ significantly from the optimal model. The null hypothesis of this test states that the model with a specific number of factors does not significantly differ from the optimal model. For one factor, the *p*-value is 0.0040, indicating a significant difference from the optimal model, suggesting that adding more factors could improve the fitting. However, for models with two or more factors, the *p*-value is 1.0000, indicating no significant difference from the optimal model. Therefore, based on the van der Voet T^2^ test, models with more than one factor are not significantly different from the optimal model, suggesting that using additional factors does not contribute to enhancing predictive performance.

The choice of four factors achieves a balance between explaining variance, predictive ability, and model simplicity. The root mean PRESS for the four-factor model is 0.7808, which is significantly lower than models with fewer factors, indicating a reduction error. Beyond the four factors, the root mean PRESS stabilizes, suggesting no further significant improvement in predictive accuracy. Thus, the use of four factors minimizes prediction error while maintaining a straightforward and interpretable model, avoiding unnecessary complexity.

The results for Q^2^ and Cumulative Q^2^ are 0.391205 and 0.8547702, respectively, indicating excellent predictive power. The addition of more factors does not significantly improve the cumulative Q^2^, suggesting that four factors are sufficient to capture most of the predictive ability. For R^2^X and Cumulative R^2^X, the values are 0.024149 and 0.554884, indicating that 55% of the variance in the independent variables (X) is explained using four factors. Similarly, for R^2^Y and Cumulative R^2^Y, the values are 0.004929 and 0.9999, demonstrating that nearly all the variance in the dependent variables (Y) is explained with four factors.

**Figure 7:**
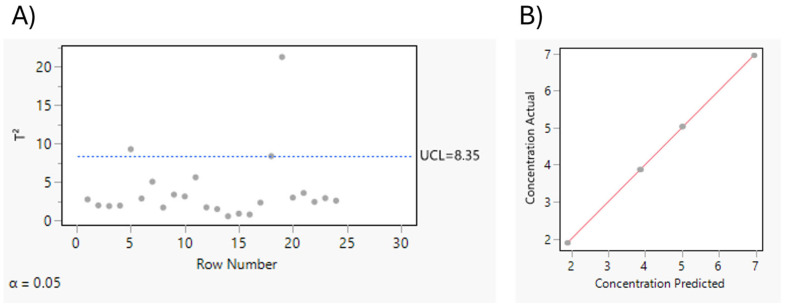
(**A**) T^2^ plot shows the Hotelling’s T^2^ statistic for each observation with control limit; (**B**) actual vs. predicted plot comparing the actual values (*y*-axis) for dependent variables.

The variable importance in projection (VIP) scores serve as a critical evaluation metric in PLS modeling, indicating the significance of each predictor variable in explaining the variance of the dependent variable (Y). When evaluating VIP, it is essential to consider the potential for overfitting. While a large number of VIP scores greater than 0.8 can suggest that more variables are effectively contributing to the model, care must be taken to avoid overfitting. Overfitting typically occurs when the model becomes overly complex (e.g., by adding too many factors), which can compromise its ability to generalize to unseen data. In this analysis, the stability of Q^2^ and root mean PRESS values with four factors indicates that the model is not overfitting. Additionally, Figure 7A, presents the T^2^ plot with a control limit, where the dashed horizontal line represents the upper control limit (UCL), set at 8.35. Observations exceeding the UCL are flagged as outliers; however, most points fall below this threshold, suggesting that the majority of the data lies within acceptable limits. Furthermore, the actual vs. predicted plot (Figure 7B) shows points that are closely clustered along the diagonal line, demonstrating a strong agreement between actual and predicted values.

In conclusion, the evaluation of the predictive NIR model against the RP-HPLC method demonstrated a strong correlation between the two approaches for bifonazole quantification. The theoretical amount of bifonazole in the preparade formulation was 10 mg/g, with RP-HPLC quantifying 8.34 mg/g and NIR predicting 8.48 mg/g. The slight underestimation compared to the theoretical value can likely be attributed to factors such as dilution and homogenization during sample preparation with the placebo formulation. Nevertheless, the relative standard deviation (RSD) of 1.25% between the RP-HPLC and NIR results underscores the reliability and precision of the NIR method as a viable alternative for rapid and accurate quantification. This highlights the potential of the NIR method for robust, non-destructive analysis in pharmaceutical formulations.

## 4. Conclusions

This study highlights the effectiveness of a partial AQbD approach streamlining early-stage development pharmaceutical research and development. NIR, combined with various chemometric algorithms, proved to be a promising tool for the non-invasive analysis of BFZ concentrations in drug formulations. By optimizing key parameters such as the number and size of subinterval, the performance of the NIR models was significantly improved. Optimized PLS models with advanced algorithms outperformed the conventional ones, highlighting NIR potential for accurate drug quantification.

The results of this study provide a strong foundation for future applications of NIR while process analytical technology in pharmaceutical analysis. Moving forward, the integration of NIR with emerging machine learning techniques could further enhance the predictive accuracy and generalizability of the models. Expanding the scope of analysis to include a wider range of drug formulations and excipients will help establish NIR as a universal method for non-destructive quantification in pharmaceutical development.

The significance of NIR lies in its capacity to facilitate rapid, non-invasive, and cost-effective analysis, a property that is especially advantageous for quality control during formulation development. Its capacity for real-time monitoring holds great potential for the streamlining of production processes and the enhancement of consistency in drug quality. In order to ensure the validity and reliability of these findings, efforts must be made to standardize NIR methodologies across and to validate their application within regulatory frameworks. This will serve to further solidify the role of NIR in the domains of drug quantification and quality assurance.

## Figures and Tables

**Table 1:** Analytical target profile (ATP) identification of a RP-HPLC and NIR method.

ATP	Target	Justification	Specification
Analyte	Bifonazole (10 mg/g)–cream	Development of RP-HPLC and NIR methods to assist bifonazole assay and permeation kinetics in the pharmaceutical formulation	N.A.
Sample type (what/where should be measured?)	Bifonazole (10 mg/g)–cream-semisolid dosage form	Method development for quantification of bifonazole in the cream pharmaceutical formulation	N.A.
Product type (when should be measured?)	Development product	Measuring spectra, provide indispensable qualitative data to assess the feasibility of manufacturing process or the final effectiveness of formulation	N.A.
Method application	Quantification of bifonazole in semisolid dosage form	The spectra profile of cream dosage form needs to be inspected, as they may influence drug delivery as well as impact patient adherence to treatment	N.A.
Analytical method	RP-HPLC analysis	Perform the measurement of the semisolid dosage form by means chromatography	N.A.
	NIR analysis	Perform the measurement of the semisolid dosage form by means spectroscopy	N.A.
Equipment	HPLC equipped with quaternary pump system and UV-Vis detector	The use of a quaternary pump allows a precise mixing of mobile-phase solvents	N.A.
	Spectrum 400 FT-IR and FT-NIR spectrometer, fitted with an InGaAs detector and an optic fiber probe with 1 mm path length	NIR is applicable to both organic and inorganic compounds, enabling its use in diverse areas	N.A.
HPLC CAAs	Number of theoretical plates, retention time, tailing factor	These attributes should meet their formal or commonly acceptable quality criteria	Number of theoretical plates: >2000; retention time: ~5.0 min; tailing factor: <2
NIR CAAs	Spectra and concentrations	These CAAs should reflect the maximization of the spectral signal	N.A.

Key: N.A.: not applied.

## Data Availability

Data will be made available upon request.
